# Chiral Materials: Multidisciplinary Progress and Emerging Frontier Application Prospects

**DOI:** 10.3390/nano15221701

**Published:** 2025-11-10

**Authors:** Feifan Xu, Hao Liu, Zhihan Jin, Tianci Huang, Chuanqi Tang, Chee Leong Tan, Yi Shi, Shancheng Yan

**Affiliations:** 1 School of Integrated Circuit Science and Engineering, Nanjing University of Posts and Telecommunications, Nanjing 210023, China; 1024223326@njupt.edu.cn (F.X.); 2023221102@njupt.edu.cn (H.L.); 1223228118@njupt.edu.cn (Z.J.); 1223228117@njupt.edu.cn (T.H.); 1024223320@njupt.edu.cn (C.T.); cheelong@gmail.com (C.L.T.); 2 National Laboratory of Solid Microstrucures, School of Electronic Science and Engineering, Nanjing University, Nanjing 210093, China

**Keywords:** chiral materials, applications, optics, electricity, quantum science, biomedicine

## Abstract

Chiral materials have shown promising application prospects across various disciplines in recent years due to their unique structural asymmetry and the resulting chiral dependence in optical, electrical, and biomedical applications. However, the existing literature lacks a unified summary of its applications in different fields. This review systematically introduces the applications of chiral materials in optics, electricity, quantum science, and biomedicine. Based on circular dichroism and chiral inversion aggregation-induced emission, chiral materials enable efficient circularly polarized light emission/detection, advancing chiral perovskite and spin light-emitting diodes. In quantum science, in-depth studies of the chiral-induced spin-selectivity effect and chiral topological superconductors support spintronic devices and quantum computing. They facilitate the development of high-efficiency energy conversion devices and high-performance chiral electrochemical sensors. In biomedicine, they excel in enantioseparation, targeted drug delivery, and theranostics. In the future, chiral materials will develop towards multi-functional integration, intelligent response, and high-performance devices. Their in-depth applications in three-dimensional display technology, low-power spin storage devices, green catalytic systems, and precision medicine will provide innovative solutions to energy, environmental, and health challenges.

## 1. Introduction

Chirality is derived from the Greek ‘χειρ’ (kheir), meaning ‘hand’, which refers to the non-overlapping nature of the object and its mirror image [1]. Like a person’s left hand and right hand, they mirror each other but cannot be overlapped entirely by rotation and translation [2]. The concept of chirality was first proposed by Louis Pasteur in 1848 when he studied tartrate crystals. He found two kinds of crystals in tartrate; their optical activities were opposite [1]. This discovery laid the foundation of stereochemistry and opened up the in-depth study of chiral phenomena. Chirality is a common phenomenon in nature. From microscopic molecules to macroscopic objects, there is a phenomenon known as chirality. At the molecular level, chirality is typically caused by asymmetric carbon atoms or specific spatial conformations [3]. Chiral molecules are called chiral molecules, and chiral molecules and their mirror isomers are called enantiomers. Enantiomers have the same physical and chemical properties, but exhibit different behaviors when interacting with a chiral environment [4,5,6].

**Figure 1:**
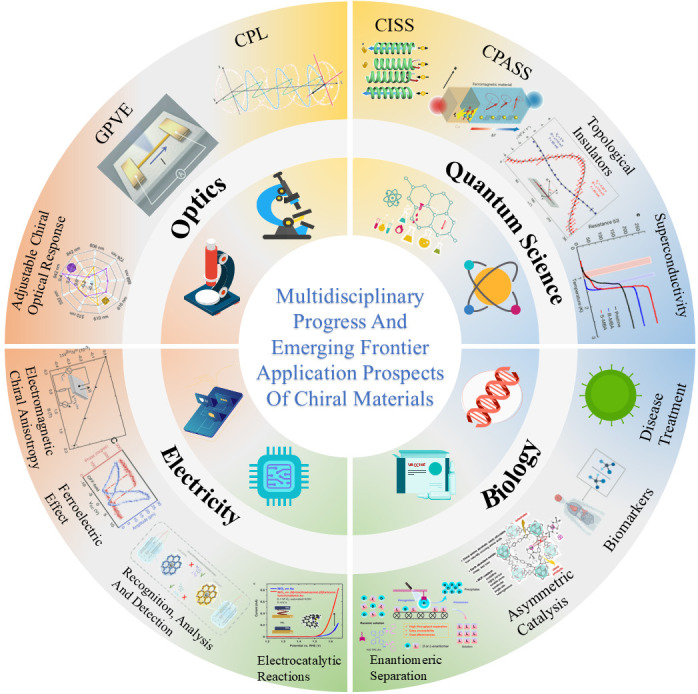
Multidisciplinary progress and emerging frontier application prospects of chiral materials [19,20,21,22,23,24,25,26,27,28,29,30,31].

The continuous emergence of new chiral materials, such as chiral metal–organic frameworks (MOFs), chiral polymers, and chiral nanomaterials, provides a richer choice for applications in various fields. The material design strategy has also extended from introducing simple chiral centers to complex systems, such as helical structures and supramolecular assemblies. At the same time, it is a current research hotspot to regulate the properties of chiral materials through physical fields (light, electricity, magnetism), chemical modification, mechanical stress, and other means, thereby achieving controllability of light, electricity, magnetism, and other responses. In addition, high-precision and high-sensitivity chiral characterization techniques, such as circular dichroism (CD) [7], vibrational circular dichroism (VCD) [8], Raman optical activity (ROA), and so on. An in-depth understanding of the relationship between the structure and properties of chiral materials provides strong support for this concept. Applying chiral materials is no longer limited to the traditional chemical field. Still, it is deeply integrated with electronics, optics, biology, medicine, and other fields, giving rise to numerous emerging cross-cutting fields. An in-depth understanding of fundamental scientific issues, such as the origin of chirality, chiral recognition, and chiral transfer, will help improve existing chemical, physical, and biological theories. Chiral materials have broad application prospects in drug synthesis [9], asymmetric catalysis [10,11], chiral separation [12], optical devices [13], biosensors [14], spintronics [15], and other fields, which can promote the development of related industries. The design and application of chiral materials can stimulate new technological innovations, such as chiral self-assembly technology, and chiral nano-manufacturing technology [16,17], and provide new ideas for solving significant challenges in energy, the environment, health, and other fields. This paper reviews the progress of applying chiral materials in electricity, optics, quantum science, and biomedicine [18], integrates the applications of chiral materials across various fields, and provides a comprehensive knowledge framework. By paying attention to the latest research progress, it reflects the frontier dynamics of chiral materials research, provides references for related fields, and promotes the innovative application of chiral materials (Figure 1). At the same time, we look forward to its future development trend.

## 2. The Application of Chiral Materials in the Field of Optics

### 2.1. Chiral Materials Emit Circularly Polarized Light

#### 2.1.1. The Definition, Classification, and Generation Mechanism of Circularly Polarized Light

Circularly polarized light (CPL) is a special kind, whose electric field vector rotates constantly in a plane perpendicular to the propagation direction. According to the rotation direction of the electric field vector, circularly polarized light can be divided into left-handed circularly polarized light (LCPL) and right-handed circularly polarized light (RCPL). From the observer’s perspective, the electric field vector rotates clockwise to the right and counterclockwise to the left [32,33]. Circularly polarized light is usually generated in two ways: first, linearly polarized light is decomposed into two beams of light whose vibration directions are perpendicular to each other, with a phase difference of π/2, using a quarter-wave plate and other delay plates. The superposition of these two beams can produce circularly polarized light [34]. Or, the chiral material has a different refractive index and absorption coefficient for left-handed and right-handed light. When light propagates through chiral materials, CD arises from exhibiting differential absorption coefficients for LCPL and RCPL. At the same time, optical rotatory dispersion (ORD) originates from the materials possessing distinct refractive indices for LCPL and RCPL—thereby inducing a discrepancy in the propagation velocities of the two polarized components. Both phenomena consequently result in CPL effect [33,35].

#### 2.1.2. The Principle of Circularly Polarized Light Emitted by Chiral Materials

The mechanism by which chiral materials emit CPL is rooted in their molecular structures and photophysical properties, and the four underlying mechanisms exhibit distinct characteristics: CD serves as the fundamental mechanism, relying on the absorption difference in ground-state chiral materials toward LCPL and RCPL circularly polarized light, and upon excitation, emits CPL that matches the material’s intrinsic chirality [36]. The molecular aggregated state regulates aggregation-induced emission chiral inversion (AIE-CI): chiral luminescent materials show weak optical activity in the single-molecule state, while intermolecular interactions can enhance or reverse chiral signals when specific molecular aggregation occurs [37]. The excited-state chirality mechanism reflects the dynamic difference between the ground and excited states of molecules. These molecules exhibit weak chirality in the ground state, but form stable helical structures upon photoexcitation, thereby emitting high-purity CPL. This enables efficient conversion of unpolarized light to CPL at the nanoscale via the interaction between precise helical geometries (e.g., chiral plasmonic nanostructures) and electromagnetic fields [38]. These mechanisms collectively constitute the theoretical basis for CPL emission by chiral materials, providing crucial guidance for the design of chiral optical materials.

#### 2.1.3. Research Status and Application Examples

In 1997, Martin et al. reviewed the research progress of new electro-optic devices and device-specific functional organic materials in the laboratory, such as optical alignment of monomer and polymer liquid crystals by linearly polarized light; optoelectronic imaging with a wide field of view of the multi-domain twisted nematic display to generate circularly polarized light display operation and compact bright cholesteric liquid crystal projection optics; the polarizer, filter, and modulator are based on liquid crystal elements. The possibility of applying circularly polarized light to display in the future is proposed [39].

**Figure 2:**
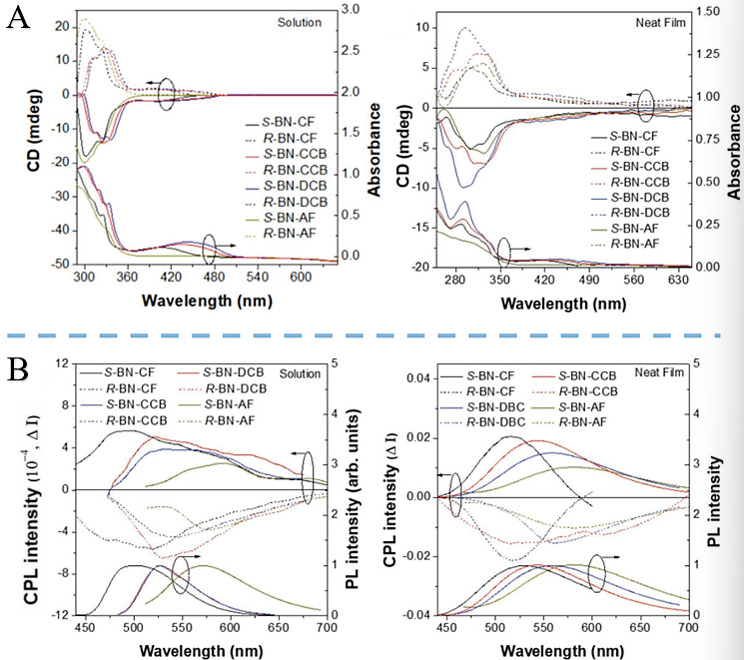
The first example of efficient CPOLEDs based on small chiral organic molecules [40]. (**A**) CD spectra of R/S-BN-CF, R/S-BN-CCB, R/S-BN-DCB, and R/S-BN-AF in toluene solution and neat film. (**B**) CPL spectra of R/S-BN-CF, R/S-BN-CCB, R/S-BN-DCB, and R/S-BN-AF in toluene and neat film state.

In 2018, Song et al. developed the first efficient CPOLEDs using small chiral organic molecules. They have overcome the key bottleneck in the integration of CPOLED material functions and the coordinated optimization of device performance. They synthesized naphthalene-containing luminescent enantiomers with AIE and delayed fluorescence. These molecules emit red/green light depending on solvent polarity (proven AIE by bright solid-state emission). All show the Cotton effect and circularly polarized luminescence in toluene solution and thin film. Multilayer CPOLEDs with doped and neat films as emitting layers achieved external quantum efficiencies of up to 9.3% and 3.5%, and gEL values of +0.026/−0.021 and +0.06/−0.06, respectively. Undoped CPOLEDs have higher gEL and smaller rol-off due to stronger AIE. Modifying the donor unit tunes electroluminescence of the doped film (493–571 nm) [40] (Figure 2).

In 2024, chiral quantum dots or chiral perovskite materials are expected to be used for circularly polarized LEDs, thereby enhancing the contrast and viewing angle of display technology. Studies have summarized the mechanism of CP electroluminescence in the most advanced materials, including organic small molecules, polymers, inorganic complexes, and hybrid halide perovskites. They discuss how to utilize the device architecture to control thermal properties and device performance, and propose improvements to maximize the efficiency and asymmetry coefficient of future CP LEDs [41]. Their research has provided a theoretical framework and a new material design paradigm for breaking through the contrast and viewing angle limitations of traditional display technologies.

Recently, Tang et al. designed chiral ionic liquid D/L-tert-butyl alanine tetrafluoroborate (D/L-TBeBF_4_) to address the scarcity of chiral perovskite ligands. Their research has achieved a breakthrough in the collaborative optimization of the design of chiral perovskite materials, the preparation of films, and the performance of devices. When preparing perovskite films via the solution method, introducing an anti-solvent into the lattice yields high-quality chiral films. This enhances crystal quality, optimizes phase composition, and imparts chiral optical properties. The green single-junction spin-emitting diode based on it shows superior performance: over 13% circularly polarized electroluminescence polarization at room temp, max brightness 3808 cd/m^2^, and EQE 12.3% [42].

**Figure 3:**
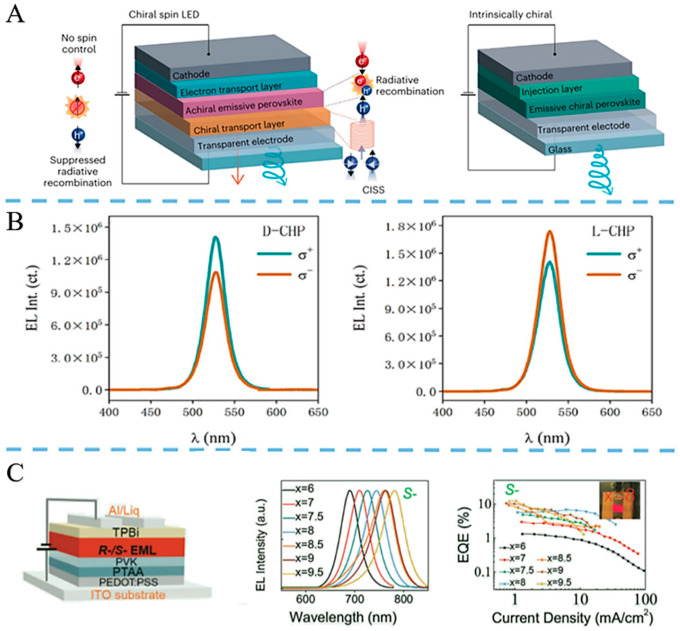
Application of circularly polarized light emitted by chiral perovskite materials. (**A**) Spin-LED architecture based on a chiral HP as a spin-selective hole-transporting layer, with radiative recombination occurring in a layer of achiral perovskite nanocrystals (left) in comparison with intrinsically chiral emissive perovskites (right). e−, electron; h+, hole [41]. (**B**) Based on D-CHP and L-CHP, spin light-emitting diodes (spin-LEDs), respectively, exhibit circularly polarized electroluminescence [42]. (**C**) The device structure and EL performance of spin-LEDs using S-NEA_2_(FA_0.8_MA_0.2_)_2_Pb_3_Br_(10−x)_I_x_ (x = 6, 7, 7.5, 8, 8.5, 9, 9.5) perovskites [43,44].

In addition, Yao et al. prepared chiral quasi-2D perovskites R/S-NEA_2_(FA_0.8_MA_0.2_)_2_ Pb_3_Br_(10−x)_I_x_ by halide composition adjustment, with emission at 675–788 nm. Chirality transfers from low to 3D perovskite via ultrafast energy transfer, inducing 3D CPL, with glum 0.0085–0.026. Passivating with multi-phosphate additives suppresses defects, resulting in PLQYs exceeding 86%. Efficient red/NIR spin-LEDs with tunable peaks are shown, with peak EQE 12.4% and gEL 0.0148 at room temp. This work links spin polarization and chiral perovskite composition in spin-LEDs, enabling new spin optoelectronic applications [43,44] (Figure 3).

### 2.2. Detection of Circularly Polarized Light by Chiral Materials

#### 2.2.1. The Principle of Detecting Circularly Polarized Light by Chiral Materials

The detection of circularly polarized light by chiral materials mainly depends on its inherent optical activity and differential response to left-handed and right-handed circularly polarized light. When circularly polarized light passes through a chiral material, the material will produce different absorption, refraction, or scattering effects on two kinds of circularly polarized light based on the chiral characteristics of its molecules or structures. Various optical methods can detect and analyze this difference [45]. For example, CD spectroscopy can be used to determine the rotation and intensity of circularly polarized light by measuring the absorption difference of chiral materials to circularly polarized light with different rotation directions [46]; in addition, the fluorescence anisotropy or CPL characteristics of chiral materials can also be used to detect circularly polarized light, because chiral materials emit circularly polarized fluorescence related to the rotation direction of incident light after being excited by circularly polarized light. The characteristics of incident circularly polarized light can be deduced by analyzing the rotation direction and intensity of the fluorescence signal [47]. For materials exhibiting chiral plasmonic effects or chiral metasurfaces, circularly polarized light can be detected by measuring the changes in polarization state of the transmitted or reflected light. Together, these detection mechanisms form the basis for applying chiral materials in circularly polarized light detection, providing sensitive and specific tools for identifying and analyzing circularly polarized light [48,49,50].

#### 2.2.2. Detection Methods and Techniques of Chiral Materials

Chiral materials’ detection methods and techniques are mainly based on their interaction with circularly polarized light, and the accurate characterization of chiral signals is achieved through various advanced optical means. CD spectroscopy is the most commonly used detection technique. The chiral optical activity of materials in the ultraviolet-visible region can be revealed by measuring the difference in the absorption of left-handed and right-handed circularly polarized light by chiral materials [46]. For chiral luminescent materials, CPL spectroscopy can directly characterize the rotation and asymmetry factor of the circularly polarized light emitted by the material, thereby evaluating its chiral luminescence properties [36]. In addition, vibrational circular dichroism (VCD) and ROA spectroscopy provide chiral information on molecular vibration modes by utilizing infrared and Raman scattering effects, respectively, which are suitable for studying the configuration and conformation of chiral molecules. At the nanoscale, the chiral plasma effect can be characterized by transmission electron microscopy (TEM) combined with electron energy loss spectroscopy (EELS) [51]. The optical properties of chiral metamaterials can be accurately measured using an ellipsometer and Mueller matrix imaging technology [52]. In recent years, ultrafast and single-molecule spectroscopy have also been employed to investigate the dynamic chiral behavior of chiral materials and their evolution on the femtosecond timescale. The comprehensive application of these detection methods and techniques provides a comprehensive characterization method for the structural analysis, performance evaluation, and application in chiral optical devices of chiral materials [53].

#### 2.2.3. Research Status and Application Examples

In 2020, Wangn et al. designed an organic chiral polymer nanowire with strong orbital angular momentum to make a circularly polarized photodetector. In chiral polymer nanowires, chirally induced orbital angular momentum leads to the splitting of spin-up and spin-down energy levels, which determines the performance of circularly polarized light detection. In addition, the circularly polarized photodetector based on chiral polymer nanowires exhibits excellent reversibility and stability after hundreds of switching operations, laying a solid foundation for potential applications [54].

In 2022, based on the excellent crystallinity and pure crystal orientation of chiral one-dimensional perovskite microwire arrays, Zhao et al. [55] fabricated a high-performance CPL detector with a maximum anisotropy factor of 0.23, a response rate exceeding 26 mA W^−1^, and a detection rate exceeding 2.2 × 10^11^ Jones. Their research has broken through the integration limitations of traditional detectors that rely on additional optical components. Moreover, the material system with environmental stability they have developed provides a brand-new practical platform for CPL detection and multi-functional devices. Meanwhile, research shows that chiral 1D perovskite single crystal microwire arrays with pure orientation and excellent environmental stability offer a potential platform for CPL detectors and other multifunctional applications [56,57,58].

**Figure 4:**
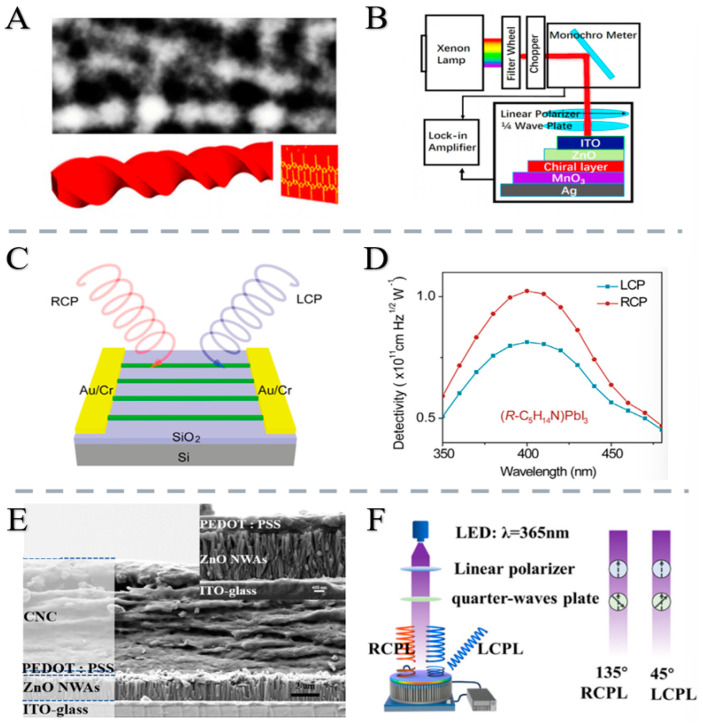
CPL photodetector. (**A**) TEM and scheme of the chiral PT nanowire structure. (**B**) Scheme of the setup of CPL detection [54]. (**C**) Schematic of the photodetector prepared with microwire arrays. (**D**) Wavelength-dependent detectivities of (R-C5H14N) Pbl3 microwire devices under different CPL illuminations [55]. (**E**) SEM image of the CPL detector cross-section. (**F**) Photoelectric test system schematic diagram of the detector [59].

In 2024, Yang et al. [59] developed a high-performance CPL detector based on CNC-ZnO nanowire arrays, exhibiting photosensitivity of 3.68, responsivity of 0.58 A W^−1^, specific detection rate of 1.29 × 10^12^ Jones, external quantum efficiency of 69.42%, fast response speed (1.53 s), and high asymmetry coefficient (0.36) that exceeds most similar studies [56,57,58]. Leveraging these properties, they proposed an ASCII coding-based optical communication method with great potential in optical communication, while the detector also effectively detects other phases of elliptically polarized light. Additionally, a simple and efficient multi-layer structure utilizing a bottom-up manufacturing method with a high success rate and a straightforward process is proposed, offering significant value for the integration, high performance, and environmental sustainability of CPL detectors [59] (Figure 4).

### 2.3. Selective Response of Chiral Materials to Circularly Polarized Light

#### 2.3.1. Selective Response of Circularly Polarized Light

The selective response and reflection of chiral materials to circularly polarized light originate from their unique spatially asymmetric structure, which leads to different optical properties for LCPL and RCPL [47]. When a circularly polarized light is incident on a chiral material, the electrons and molecular structures in the material interact with the electromagnetic field of the light. Due to the helical or asymmetric arrangement of the chiral material, the propagation speed and absorption characteristics of the left-handed and right-handed circularly polarized light will be different. This phenomenon is called CD [45]. Specifically, the response of electronic transitions and molecular vibration modes in chiral materials to LCPL and RCPL differs, resulting in one circularly polarized light being more strongly absorbed or reflected than the other. At the same time, the other may be transmitted or scattered [46]. In addition, the refractive index of chiral materials differs for LCPL and RCPL (known as optical rotational dispersion), which causes the polarization direction of circularly polarized light to rotate as it propagates through the material. These selective response and reflection characteristics are particularly significant in materials such as chiral photonic crystals, chiral metamaterials, and chiral liquid crystals [60,61,62,63,64,65,66], providing an essential basis for applications such as circularly polarized photodetectors [56,57,58], optical filters, and chiral sensing.

#### 2.3.2. Research Status and Application Examples

Ai et al. proposed an integrated strategy for designing and synthesizing ternary optical switches. Phosphorescent cyclometalated platinum and photochromic spiropyran were used as triplet sensitizers and optical switch construction modules, and the bridge was realized by chiral cyclohexyldiamine. Significant research progress has been made in the dynamic regulation of visible light, advanced information storage, and anti-counterfeiting [67].

**Figure 5:**
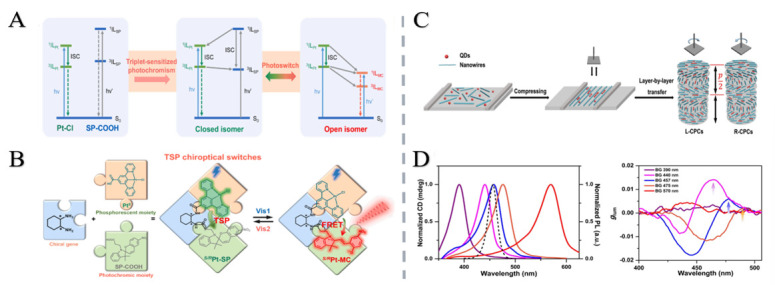
The application of chiral materials in selective response to circularly polarized light. (**A**) Illustration of the mechanism for the visible light-controlled molecular switches by TSP. (**B**) The molecular design strategy of the photoreversible CPL switches of S/RPt-SP [67]. (**C**) Scheme of fabricating QD-doped CPCs through layer-by-layer Langmuir–Schaefer co-assembly of colloidal nanowires and QDs. (**D**) Modulation of luminescence polarization [74].

The chiral photonic crystal is a photonic material featuring a spiral or mirror-asymmetric periodic structure. Its unique spatial arrangement enables it to form a photonic band gap, meaning that light in a specific frequency range cannot propagate through it [68,69]. Due to the coupling between the chiral structure and the spin angular momentum of the circularly polarized light, the left and right circularly polarized light will experience different effective refractive indices in the crystal, resulting in a shift in the photonic band gap positions of the two [15,70]. When the frequency of the incident light is within the band gap of a specific direction (such as right rotation), the light in this direction is strongly reflected by Bragg scattering. In contrast, the light in the other direction (left rotation) can be transmitted because the frequency is outside the band gap. This selective reflection originates from the spin-dependent modulation of circularly polarized light by a chiral structure, providing a new way to dynamically regulate the light polarization state [71,72,73]. Recently, based on previous studies, Tang et al. used ultrafine NiMoO_4_·xH_2_O nanowires and CdSSe@ZnS quantum dots as building blocks to construct an inorganic chiral photonic crystal with one-dimensional helical structure by the Langmuir–Schaeffer technique, which realized the precise control of the sign, position, and intensity of the CPL peak [74] (Figure 5).

### 2.4. Chiral Materials Have a Giant Photovoltaic Effect

#### 2.4.1. Definition of Giant Photovoltaic Effect

The giant photovoltaic effect refers to the phenomenon of some materials producing abnormally high photo-generated voltage or photo-generated current under light conditions, which is much stronger than the photoelectric conversion efficiency of traditional photovoltaic materials (such as silicon-based solar cells). This effect is usually closely related to the special electronic structure, interface characteristics, or symmetry breaking in materials.

#### 2.4.2. Giant Photovoltaic Effect in Chiral Materials

In chiral materials, the giant photovoltaic effect exhibits unique chiral dependence, that is, the response of materials to left-handed and right-handed circularly polarized light is significantly different. This difference is due to the inherent structural asymmetry of chiral materials, which enables the induction of a stronger spin-polarized current or charge separation efficiency when circularly polarized light interacts with the material [74]. For example, circularly polarized light in chiral perovskite materials or chiral molecular crystals can excite the material to generate photo-generated carriers related to the optical rotation, thereby achieving efficient photoelectric conversion based on chiral selectivity [75]. In addition, the helical structure or chiral interface in chiral materials can also enhance the separation and transmission efficiency of photogenerated carriers, further amplifying the photovoltaic effect. This chiral-dependent giant photovoltaic effect not only provides a new idea for designing efficient photoelectric conversion devices, but also opens up a new research direction for the development of spintronics and chiral optoelectronics.

#### 2.4.3. The Physical Mechanism of the Giant Photovoltaic Effect in Chiral Materials

The physical mechanism of the giant photovoltaic effect of chiral materials is mainly due to its unique structural asymmetry and chiral-dependent light–matter interaction. Firstly, the helical structure or molecular chiral center of chiral material breaks the space inversion symmetry of the material, so that when the material is illuminated by circularly polarized light, it can produce spin-polarized carriers related to the rotation direction of light. This spin-selective charge separation process significantly enhances the photovoltaic effect [74,76]. Secondly, CISS effect in chiral materials plays a key role in the photoexcitation process: the interaction between circularly polarized light and chiral molecules leads to the spin polarization of photogenerated electrons and holes, and forms a spin-related band structure inside the material, thereby promoting efficient charge separation and transport [77]. In addition, the chiral interface or chiral superstructure in chiral materials can generate an internal electric field, further enhancing the separation efficiency of photogenerated carriers and inhibiting their recombination. At the same time, the nonlinear optical properties of chiral materials may also induce higher photoelectric conversion efficiency under a strong light field [78]. These mechanisms work together to enable chiral materials to produce a significant photovoltaic effect, far exceeding that of traditional photovoltaic materials, under circularly polarized light irradiation. This provides a theoretical basis for developing efficient chiral optoelectronic devices.

#### 2.4.4. Research Status and Application Examples

In 2019, Osterhoudt et al. [79] demonstrated a large mid-infrared bulk photovoltaic effect (BPVE) in Weyl semimetal TaAs microdevices, integrating Weyl semimetal research, focused ion beam fabrication, and theoretical modeling. Optimized device design minimized thermal effects and resistance losses during photocurrent measurement, while symmetry analysis effectively separated displacement current from photothermal effects, as verified by both experiments and calculations. The results showed that TaAs BPVE exhibited a strong mid-infrared response, with a Glass coefficient nearly an order of magnitude higher than the recently reported giant BPVE in BaTiO_3_, opening a new energy range for BPVE and Weyl semimetal applications [76,80].

In 2024, Wang et al. [81] reported the giant infrared bulk photovoltaic effect (BPVE) in tellurium (Te). BPVE causes the photocurrent under uniform illumination. The wavelength range of bulk photovoltaic in Te is from ultraviolet to mid-infrared. Its photocurrent density under infrared light is better than that of previous materials. Neurons attached to cortical neurons can induce action potentials under broad-spectrum light [82]. This work lays the foundation for the further development of infrared BPVE in narrow band-gap materials.

**Figure 6:**
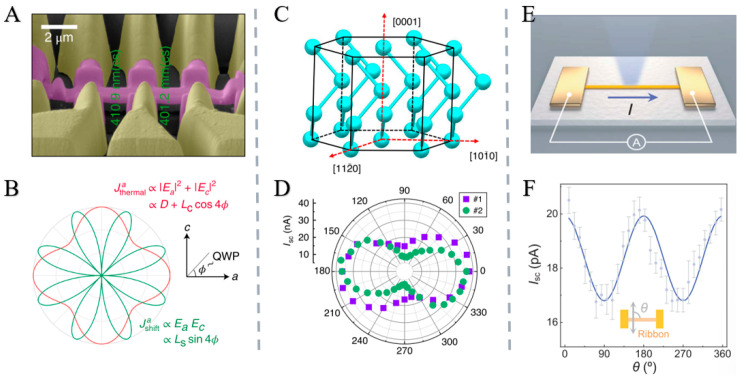
Giant photovoltaic effect in chiral materials. (**A**) False-color scanning electron microscopy (SEM) image of a microscopic TaAs (purple) device with Au (yellow) contacts. (**B**) Polarization dependence of thermal ($J_{thermal}^{a}$) and shift ($J_{shift}^{a}$) photocurrent contributions [79]. (**C**) Schematic of the atomic structure Te. (**D**) Infrared linear polarization dependence of Isc in Te devices [81]. (**E**) Schematic of the ribbon-based BPVE device. (**F**) Polarization-dependent Isc of AC-R with ~150 nm width [19].

In addition, Liu et al. synthesized single-crystal WS_2_ ribbon arrays with tunable chirality and coherent polarity via atomic manufacturing. Their research has made a breakthrough in overcoming the collective output problem of 1D-TMD devices, and has provided a key material system for exploring solar energy collection beyond the SQ limit of p-n junctions by utilizing enhanced BPVE. Chirality is defined by substrate coupling, while interfacial energy determines polar direction. Armchair ribbons exhibit robust BPVE, and integrating approximately 1000 aligned ribbons amplifies the photocurrent. This overcomes collective output challenges in 1D-TMD devices, leveraging enhanced BPVE to explore exceeding the SQ limit in p-n junction solar harvesting [19,83] (Figure 6).

### 2.5. Chiral Materials Achieve an Adjustable Chiral Optical Response

Cen et al. [83] jointly demonstrated a microfluidic hybrid emission system consisting of a suspended twisted stacked metasurface coated with PbS quantum dots. The suspended metasurface is prepared using one-step electron beam lithography, exhibiting a strong optical chirality of 309 °μm^−1^ at key spectral positions and a thickness of less than λ/10. Through a significant chiral-selective interaction, photoluminescence is enhanced and exhibits strong asymmetry in circularly polarized light [84,85,86]. The hybrid system induces circularly polarized luminescence with an asymmetry factor of 1.54. Modulating the metasurface substrate’s refractive index enables a tunable chiral optical response and reversible chiral inversion emission. This active hybrid architecture shows promise for anti-counterfeiting, biosensing, polarized light sources, imaging, and optoelectronic displays [40].

**Figure 7:**
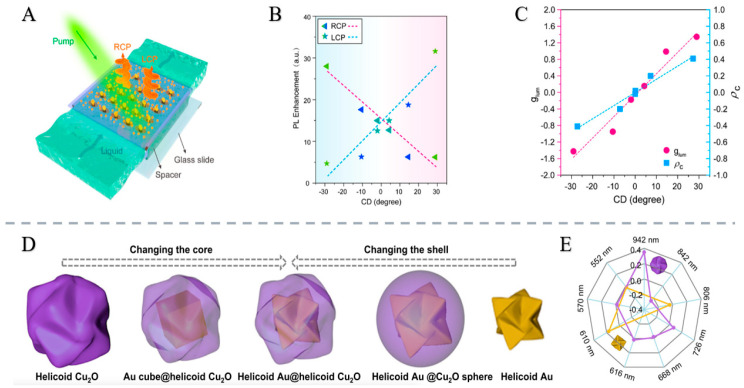
Chiral materials achieve adjustable chiral optical response. (**A**) Schematic diagram of PL investigation from QDs–metasurface–liquid composites. (**B**) PL enhancements of the RCP and LCP emission on enantiomer A and enantiomer B as a function of CD values at the wavelength of the emission peak. (**C**) Measured glum and correspondingly simulated ρc of hybrid systems with active mediums as a function of CD values at the wavelength of the emission peak [83]. (**D**) The geometrical models of the helicoid Cu_2_O nanoparticles, the Au cube@helicoid Cu_2_O nanoparticles, the helicoid Au@helicoid Cu_2_O nanoparticles, the helicoid Au@Cu_2_O sphere nanoparticles, and the helicoid Au nanoparticles. (**E**) The comparison of g-factor values and corresponding wavelengths of the D-handed helicoid Au (yellow dots) and the D-handed helicoid Au@Cu_2_O-6 (purple dots) nanoparticles, which is a helical variant of Au@Cu_2_O with different Cu_2_O thicknesses, and the molar ratio of Cu_2_O to Au is 1.09 [26].

In addition, Lv et al. [26] proposed a chiral plasmon–dielectric coupling strategy to extend the chiral optical response of helical Au@Cu_2_O nanoparticles to the near-infrared region (Figure 7). Epitaxially synthesized helical Au@Cu_2_O nanoparticles with intrinsic structural chirality exhibit a g factor of up to 0.35. Strong coupling between the high-refractive-index Cu_2_O shell and chiral plasmonic Au core induces enhanced electric/magnetic multipole resonances, enabling pronounced chiral optical responses. These nanoparticles demonstrate significant polarization rotation capability, holding promise for applications in anti-counterfeiting, encryption, and chiral sensing [87,88].

### 2.6. The Applications of Chiral Liquid Crystal Materials

Chiral liquid crystal materials are functional materials that encompass chiral characteristics and liquid crystalline order. Their core principle resides in the synergistic coupling effect between the chiral structure at the molecular/supramolecular level and the long-range orientational order as well as positional disorder inherent to the liquid crystalline state. Chirality induces molecules to form helical supramolecular structures, typically exemplified by the cholesteric phase, whose pitch exhibits significant sensitivity to external stimuli. Endowed with this characteristic structure, the materials not only demonstrate typical chiral optical effects such as circular dichroism and optical rotation but also possess excellent stimulus responsiveness along with chiral recognition and separation capabilities, laying a core foundation for their diverse relevant applications in multiple fields.

To address the intrinsic limitations of conventional chiral liquid crystal materials, which are characterized by the challenge of achieving complete chirality inversion and the micrometer-scale resolution bottleneck in structured device fabrication, in 2025, Li et al. [89] proposed a bilayer chiral framework integrating nanokirigami technology with self-assembly methodologies. This framework incorporates nanocilia structures with cholesteric liquid crystals (CLCs) and enables the achievement of dynamic amplification, abrogation, and inversion of optical chirality through the precise regulation of the static optical chirality of nanocilia and the dynamic optical chirality of CLCs. Capitalizing on this bilayer chiral architecture, a thermally tunable circularly polarized light metal lens has been developed. This achievement overcomes the geometric constraints of traditional nanokirigami structures and furnishes a novel high-performance platform for the dynamic modulation of optical chirality in the fields of optical communication and display technologies.

Recently, Sun et al. [90] proposed a touch-mediated dynamic nested optical encryption scheme leveraging touch-driven cholesteric liquid crystal (CLC) superstructures. As the core of this scheme, it integrates relief-structured CLCs with thermosensitive reverse chiral CLCs, thereby enabling the realization of multi-dimensional optical information encryption. This study has overcome the dynamic regulation bottleneck of traditional optical encryption technologies, furnished a novel paradigm for high-security and high-capacity optical information processing, and facilitated the on-demand construction of chiral nanostructures as well as the advancement of smart-responsive materials.

## 3. The Application of Chiral Materials in the Field of Quantum Science

### 3.1. Chiral-Induced Spin Selectivity (CISS)

#### 3.1.1. The Origin and Research Development of the CISS Phenomenon

The origin of the CISS (chiral-induced spin selectivity) phenomenon can be traced back to the study of the interaction between chiral molecules and spin-polarized electrons at the end of the 20th century. Still, its systematic research began in the early 21st century [91,92]. In 2004, Naaman et al. first observed in an experiment that electrons exhibit significant spin selectivity when passing through chiral molecules, specifically that electrons with a particular spin direction are more likely to pass through them. This discovery marks the formal presentation of the CISS effect [93]. Subsequent studies have shown that the CISS effect originates from the interaction between the structural asymmetry of chiral molecules and the spin–orbit coupling of electrons. When electrons pass through chiral molecules, the helical structure of molecules will couple with the spin angular momentum of electrons, resulting in a significant increase in the probability of electron transport in a specific spin direction [94]. Significant progress has been made in studying the CISS effect in recent years. For example, the effects of chiral molecular length, helicity, and electronic structure on spin selectivity have been revealed at the molecular scale, and the universality of the CISS effect has been verified in nanomaterials, biomolecules, and interface systems [95]. In addition, the CISS effect is also widely used in spintronics, chiral catalysis, and quantum information, providing an essential basis for developing new spin devices and exploring the fundamental physical principles of chiral–spin interaction [96].

#### 3.1.2. The Theoretical Basis of CISS

The theoretical basis of CISS is mainly based on the interaction between the structural asymmetry of chiral molecules and the spin–orbit coupling (SOC) [93]. When an electron passes through a chiral molecule, the helical structure of the molecule will break the spatial inversion symmetry. At the same time, the electron’s motion is associated with the spin angular momentum through spin–orbit coupling, resulting in different scattering potentials for electrons in a specific spin direction during transmission. This asymmetric scattering potential facilitates the passage of electrons in one spin direction through chiral molecules. In contrast, electrons in the other spin direction are suppressed, resulting in a spin-selective transmission effect. Theoretical studies have shown that the strength of the CISS effect is closely related to the helicity, length, and details of electron–molecule interactions in chiral molecules [97,98,99,100]. In addition, the electric field gradient in chiral molecules will further enhance the spin–orbit coupling through the Rashba effect, thereby amplifying the spin selectivity. These theoretical frameworks explain the CISS effect’s experimental observations and provide theoretical guidance for predicting and designing chiral materials with high spin selectivity [101,102,103].

#### 3.1.3. Research Status and Application Examples

In 2013, Ben Dor et al. [104] combined the CISS effect with the STT-RAM concept to demonstrate a spin memory technology that does not require magnets. Leveraging this effect, a concept for a permanent magnet-free chiral magnetic silicon-based universal memory device compatible with CMOS technology is demonstrated. Specifically, spin-selective charge transfer via self-assembled polyalanine monolayers magnetizes the Ni layer, achieving a remanent magnetization equivalent to an external magnetic field of 0.4 T. Data reading is realized through low-current operations, positioning this technology to overcome limitations of conventional magnetic storage and enable low-cost, high-density on-chip universal memory devices [105,106,107].

**Figure 8:**
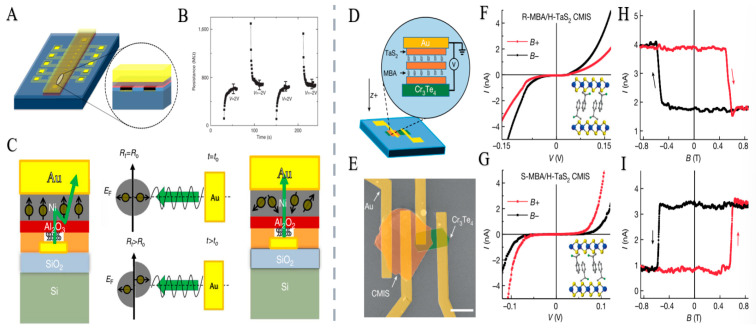
Chiral-induced spin selectivity. (**A**) Device scheme. (**B**) Memory effect. (**C**) Schematic drawing of the experimental concept [104]. (**D**) Schematic drawing of a typical CMIS STJ device. (**E**) A false-colored SEM image of a typical device. (**F**,**G**) I–V characteristics of a R-MBA/H-TaS_2_ CMIS. (**H**) Magnetic-field-dependent tunneling current measured in the R-MBA/H-TaS_2_ CMIS. (**I**) Magnetic-field-dependent tunneling current measured in the S-MBA/H-TaS_2_ [20].

In recent years, although researchers have developed several methods to introduce CISS into solid-state material systems, the high inhomogeneity often plagues these preparation methods and their material systems, low spin selectivity, and limited stability of chiral molecular systems, and it is difficult to finally form spintronic devices with high selectivity and high stability [105,106,107]. Given the above key problems that need to be solved urgently, Qian et al. [20], this work expands the solid-state material system for CISS research by developing a novel high-quality chiral molecular intercalated superlattice (CMIS) material. The CMIS features a highly ordered superlattice structure with chiral optical selectivity. Leveraging CMIS as an electron spin filter layer, the authors designed efficient spin-tunneling devices that exhibit a spin reluctance ratio exceeding 300% and a spin polarization exceeding 60% (Figure 8).

### 3.2. Chiral Materials Have Chiral-Phonon-Activated Spin Seebeck Effect (CPASS)

#### 3.2.1. The Origin of the CPASS Phenomenon

The spin Seebeck effect (SSE) is a core phenomenon in the field of spintronics, referring to the phenomenon of spin current driven by a temperature gradient. Its physical essence lies in the coupling between spin angular momentum and lattice vibrations (phonons). Traditional SSE mainly studies the spin transport in ferromagnetic materials, while Chiral-phonon-activated spin Seebeck effect (CPASS) in chiral materials introduces the unique properties of chiral structures [108]. Due to the spatial symmetry breaking, the lattice vibration mode (chiral phonon) of chiral materials (such as helical crystals and chiral molecular crystals) has intrinsic angular momentum, which provides a new mechanism for generating and regulating spin current.

#### 3.2.2. The Theoretical Basis of CPASS

The theoretical framework of CPASS is based on the following core mechanisms: first, the lattice vibration mode of chiral materials (chiral phonons) carries non-zero orbital angular momentum, which is converted into electron spin polarization by spin–orbit coupling (SOC). Secondly, the temperature gradient leads to the non-equilibrium distribution of chiral phonons, and its angular momentum is transmitted to the local magnetic moment or conduction electrons through magnetoelastic coupling, resulting in spin. Finally, the breaking of the space group symmetry of chiral materials (the lack of mirror symmetry) allows the directional transport of spin current in a specific direction (along the direction of temperature gradient), which is different from the isotropic characteristics of traditional SSE [109,110].

#### 3.2.3. CPASS-Related Specific Applications

The CPASS in chiral materials has shown vast application potential in many fields. In spintronics devices, the spin current generated by CPASS can replace the traditional charge current to develop low-power spin logic devices (such as spin field-effect transistors), which can significantly reduce energy consumption. At the same time, a chiral thermoelectric converter based on a temperature gradient to generate spin-polarized current directly can be used for highly sensitive thermoelectric sensors [111,112]. In quantum computing and information storage, CPASS regulates the spin quantum state through chiral phonons, enabling the rapid initialization and manipulation of spin qubits. It utilizes thermally induced spin current to drive the movement of magnetic domain walls, thereby improving the storage density and speed of non-volatile memory [113]. In addition, in terms of energy collection and conversion, the CPASS-driven spin thermoelectric generator can efficiently convert waste heat into electrical energy, with a conversion efficiency more than 30% higher than that of traditional thermoelectric materials. This provides a new approach to clean energy technology. These applications not only promote the development of spintronics, but also bring innovative breakthroughs in the field of quantum technology and energy.

#### 3.2.4. Research Status and Application Examples

**Figure 9:**
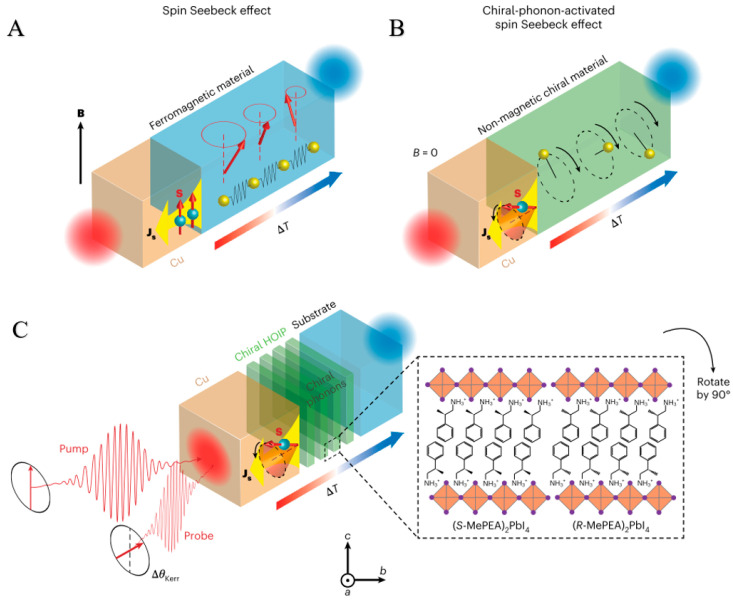
Chiral materials have CPASS effect. (**A**) Schematic illustration of the CPASS effect. (**B**) Schematic illustration of the CPASS effect. (**C**) Using the TR-MOKE technique, the measurement geometry (not to scale) of the CPASS effect in layered Cu/chiral HOIP heterostructures is used [21].

Kim et al. have discovered a new type of spin selection effect, known as the CPASS effect, which occurs without a magnetic field and is excited by chiral phonons, characterized by strong spin–orbit coupling. Based on the non-equilibrium chiral phonon distribution under a temperature gradient, the CPASS effect strength was calculated using the Boltzmann equation, which analyzes the contributions of electron band transport and phonon drag. Results show that spin accumulation scales with the temperature gradient squared, modulated by the chemical potential in total strength and component ratios. This CPASS effect elucidates spin selection in traditional chiral systems, enabling the development of novel chiral-material-based spintronic devices [21] (Figure 9).

### 3.3. Chiral Materials Can Be Used to Make Topological Insulators

Chiral materials can induce strong spin–orbit coupling effects in materials by introducing chiral molecules or chiral nanostructures, thereby promoting the formation of topologically nontrivial energy bands. For example, chiral organic molecules or metal–organic frameworks (MOFs) can form crystal structures with helical symmetry through self-assembly. This structure can break the spatial inversion symmetry and enhance the spin–orbit interaction, thereby achieving topological insulation [114,115]. In addition, the CISS effect in chiral materials can further regulate the spin polarization characteristics of surface states, making them potentially applicable in low-energy spintronic devices and quantum computing. By rationally designing the molecular configuration and crystal arrangement of chiral materials, new chiral topological insulators can be prepared, providing a new material platform for exploring topological quantum phenomena and developing high-performance electronic devices [116].

**Figure 10:**
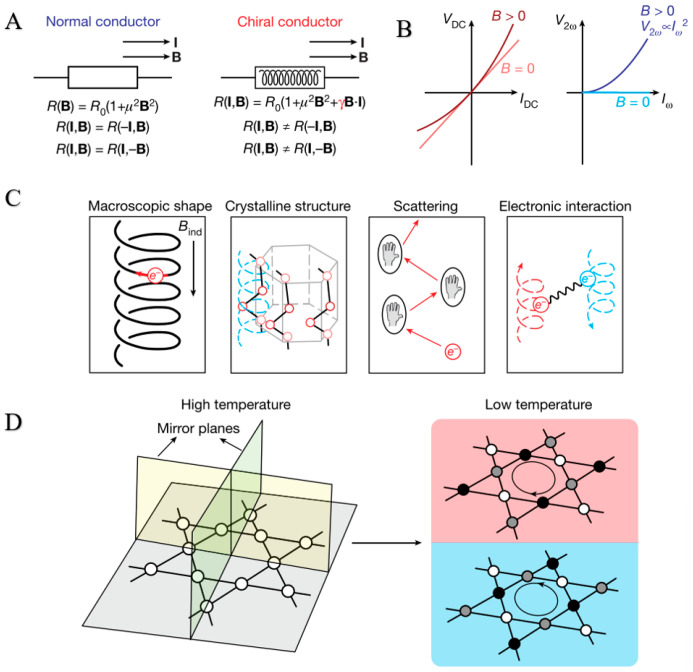
Chiral materials can be used to make topological insulators. (**A**) Illustration of the electrical resistance of regular and chiral conductors within the low-frequency limit. (**B**) I (V) curve for a chiral conductor. (**C**) Different mechanisms for electronic magnetochiral anisotropy. (**D**) The crystal structure of CsV3 Sb5 preserves all mirror symmetries at high temperatures and only spontaneous symmetry breaking at low temperatures enables a finite eMChA in a symmetric microstructure [117].

Electromagnetic chiral anisotropy is an essential characteristic of chiral electronic materials. Similarly to the diode effect, it describes that the current’s direction directly affects the material’s resistance due to the symmetry breaking of the mirror. So far, this effect has only been observed in materials with a chiral crystal structure [22]. Recently, Guo et al. first observed this effect in the Kagome superconductor CsV_3_Sb_5_, a material with a centrosymmetric, achiral lattice structure [117] (Figure 10).

### 3.4. Chiral Superconductivity

#### 3.4.1. Chiral Superconductors

Due to their unique structural and electronic properties, superconducting materials have demonstrated significant potential in achieving high-temperature superconductivity. By breaking the spatial inversion symmetry, the chiral structure can induce strong spin–orbit coupling and unconventional pairing mechanisms, thereby enhancing the formation and stability of superconducting states. For example, helical symmetry or chiral molecular alignment in chiral crystals can facilitate unconventional p-wave or d-wave superconducting pairings, which typically have higher critical temperatures [23,118]. In addition, the CISS effect in chiral superconducting materials can further regulate the electron spin state, enhance the condensation ability of Cooper pairs, and suppress the destruction of superconducting states by thermal fluctuations [119,120]. Experimental studies have shown that some chiral organic superconductors and chiral metal–organic frameworks (MOFs) exhibit superconducting properties at relatively high temperatures, providing a new research direction for exploring the mechanism of high-temperature superconductivity [121]. By rationally designing the molecular configuration and crystal structure of chiral materials, their superconducting properties can be optimized, paving the way for achieving the goal of room-temperature superconductors. The study of chiral superconducting materials deepens our understanding of the superconducting mechanism and provides an important material basis for the development of efficient energy transmission and quantum computing technology [122,123,124].

**Figure 11:**
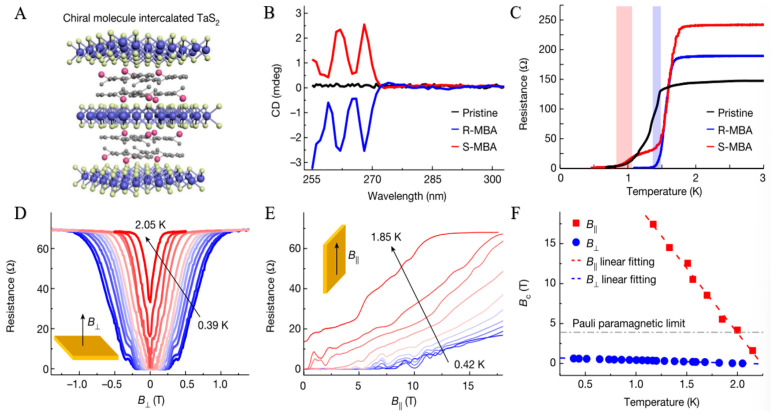
Chiral superconductors. (**A**) Schematic drawings of a chiral molecule intercalated in 2H–TaS_2_. (**B**) CD spectra of right-handed methylbenzylamine (R-MBA) and left-handed methylbenzylamine (S-MBA) chiral molecules intercalated and pristine 2H–TaS_2_. (**C**) Resistance of pristine, R-MBA and S-MBA intercalated 2H–TaS_2_ as a function of temperature. (**D**) The out-of-plane magnetic field dependence of resistance at different temperatures. (**E**) In-plane magnetic-field dependence of the resistance under different temperatures. (**F**) Extracted in-plane upper critical field and out-of-plane upper critical field as a function of temperature [23].

In 2024, Wan et al. reported an innovative method to induce unconventional superconductivity by introducing chiral molecules into traditional superconductor lattices [23]. The unconventional superconductors, known as chiral superconductors, whose superconducting order parameters are wound clockwise or counterclockwise in momentum space, exhibit topologically nontrivial properties and time-reversal symmetry-breaking characteristics, making them promising for applications in topological quantum computing (Figure 11).

#### 3.4.2. Chiral Topological Superconductors

In 2024, Ning et al. [125], proposed the Floquet project of a practical two-dimensional topological nodal superconductor composed of antiferromagnetic monolayers of neighboring s-wave superconductors. Breaking light-induced effective time-reversal symmetry enables Floquet chiral topological superconductivity, tunable via elliptically polarized light. Photon-modified spectra exhibit distinct Chern numbers, with topological transitions driven by the generation and annihilation of valley pairs resulting from the interplay between magnetic symmetry, superconductivity, and topology. This provides a viable route to dynamically tunable Floquet chiral topological superconductivity, which merits experimental focus [126,127,128,129,130,131].

## 4. The Application of Chiral Materials in the Field of Electricity

### 4.1. Chiral Materials Have Electromagnetic Chiral Anisotropy

#### 4.1.1. Electromagnetic Chiral Anisotropy

Electromagnetic chiral anisotropy refers to the asymmetry in the optical and electromagnetic properties of materials when electromagnetic waves propagate in a specific direction and have a particular polarization state under the influence of electromagnetic fields. This anisotropy stems from the chiral characteristics of the material’s internal structure, resulting in left-handed and right-handed circularly polarized light experiencing different refractive indices, absorption rates, or phase delays during propagation [132]. This phenomenon is common in chiral metamaterials, liquid crystals, and some biomolecules. Electromagnetic chiral anisotropy is in applications such as optical device design, negative refractive index metamaterials, polarization control, and biosensing. For example, it can be used to develop efficient circularly polarized photodetectors or enhance the detection sensitivity of chiral molecules [60,61,62,63,65].

#### 4.1.2. Application Example

Recent studies have demonstrated that the superconductor CsV3Sb5 exhibits a Kagome lattice structure. As a counterpart to graphene’s more prevalent hexagonal tiling, the kagome lattice (trihexagonal tiling) emerges as a pivotal model system for exploring the emergence of unconventional electronic and magnetic properties. Traditional electromagnetic chiral anisotropy typically occurs in materials with a chiral crystal structure, as the chiral structure disrupts mirror symmetry, influencing the current direction and resistance value (similar to the diode effect). Xiang et al. first observed this phenomenon in the Kagome superconductor CsV_3_Sb_5_ [133] and published their findings. They found that in CsV_3_Sb_5_, the appearance of the charge-ordered phase leads to electromagnetic chiral anisotropy, and this chiral transport can be switched by applying external conditions (such as electric or magnetic fields). This phenomenon may be related to the formation of the charge-ordered phase in CsV_3_Sb_5_, which destroys the local symmetry and leads to electromagnetic chiral anisotropy. The specific mechanism needs further study to clarify. This finding challenges the traditional chirality cognition, indicating that even in the lattice structure with central symmetry, electromagnetic chiral anisotropy may be generated through other mechanisms (such as charge ordering). This provides a new idea for designing chiral electronic materials [134,135,136].

Zhang et al. proposed a virtual polarizer concept that leverages coherent perfect absorption and coherent perfect transparency principles to achieve polarization control of chiral electromagnetic wave. The virtual polarizer structure realizes ultra-broadband polarization manipulation via coherent interaction and interference between signal and control electromagnetic waves. For incident polarized waves with electric fields parallel to the x-y plane—separable into x-axis transverse electric and y-axis transverse magnetic components—the design achieves coherent perfect transparency transparent transmission over 0~2.31 THz and ultra-broadband coherent perfect polarization conversion across 2.72~8.41 THz (102% relative bandwidth). By modulating the indium antimonide (InSb) layer, VP enables precise control of the phase difference and polarization chirality, with high polarization conversion efficiency, thereby manipulating the propagation of EW [137,138].

### 4.2. Chiral Materials Have a Ferroelectric Effect

#### 4.2.1. Ferroelectric Effect

The ferroelectric effect is the phenomenon in which specific materials undergo polarization under an applied electric field, retain that polarization after the field is removed, and allow reversal of the polarization direction under an electric field of opposite polarity. Its fundamental mechanism stems from the non-centrosymmetric crystal structure: in ferroelectric crystals, the charge centers of positive and negative ions fail to coincide, endowing unit cells with intrinsic dipole moments that are randomly distributed at the macroscopic scale, resulting in no initial overall polarization of the material [139,140]. The application of an external electric field aligns these dipole moments, forming a macroscopic polarization. Notably, ferroelectric materials possess multiple energy-equivalent polarization directions (governed by crystal symmetry), and the dipole moments are capable of switching among these directions. After the electric field is removed, the dipole moments remain at the nearest energy minimum due to interactions between internal ions, thereby maintaining the polarized state [141,142].

#### 4.2.2. Ferroelectric Effect in Chiral Materials

Chiral ferroelectric materials derive ferroelectricity from a non-centrosymmetric crystal structure and chiral symmetry breaking. Their pivotal advantage over conventional counterparts lies in the unique spinoelectric effect, which enables modulation of the electronic structure via chiral–orbit/spin coupling, enriching polarization dynamics. They excel in multifunctional integration, holding great potential in optoelectronics, spintronics and high-density memory, while advancing the theoretical design of novel functional materials [143,144,145,146].

#### 4.2.3. Research Status and Application Examples

Han et al. [147] successfully constructed a ferroelectric vortex domain structure and introduced optical chirality by doping La3+ Ions into BiFeO_3_ nanoislands to regulate electrostatic energy. Using optical second harmonic circular dichroism (SHG-CD) and piezoelectric force microscopy (PFM), this study revealed a correlation between chiral signals and ferroelectric vortex domain structures. It demonstrated reversible non-volatile control of ferroelectric vortices via external electric fields, enabling the selective generation, and elimination of chiral signals. These findings contribute to understanding the chiral origin of inorganic ferroelectrics and lay a foundation for novel chiral optoelectronic devices [148].

**Figure 12:**
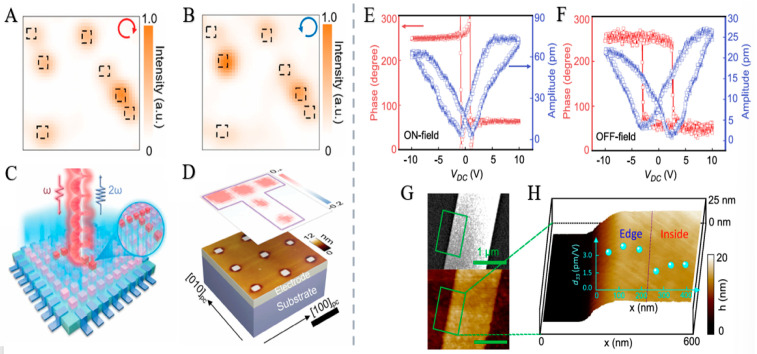
Chiral materials have a ferroelectric effect. (**A**,**B**) SHG mappings acquired with (**A**) LCP and (**B**) RCP light from the LBFO nanoislands. (**C**) Schematic illustration of chiroptoelectronic devices based on the LBFO nanoislands with a topologically chiral vortex domain. (**D**) Topography and SHC-CD images of a self-assembly, ordered LBFO nanoislands array, which displays a “T” symbol of SHG-CD signals after selective electric field polarization [147]. (**E**,**F**) Phase–voltage hysteresis loops and amplitude–voltage butterfly loops measured by vertical ON-field (**E**) and OFF-field (**F**) PFM. (**G**) Upper and lower: SEMand AFM topographical images of a Te nanosheet (height of ~15 nm) on an Au-coated silicon wafer. (**H**) Values of d33 at the different marked positions (overlaid on an AFM topograph) of the Te nanosheet [24].

Zhang et al. [24] attributed ferroelectricity to the ion displacement caused by the interlayer interaction of lone pair electrons. Ferroelectric polarization induces strong field-effect transport along Te chains, enabling self-gated ferroelectric field-effect transistors (Fe-FETs) (Figure 12). Using ferroelectric Te nanowires as channels, these devices exhibit high carrier mobility (220 cm^2^/V·s), continuous resistive switching, long data retention (greater than 10^5^ s), and ultrahigh storage density (1.92 TB/cm^2^). This work paves the way for single-element ferroelectrics, enabling applications in ultrahigh-density data storage and memcomputing devices [149].

### 4.3. Chiral Materials Are Used for Chiral Recognition, Analysis, and Detection

#### 4.3.1. Chiral Recognition

Chiral recognition refers to the ability to distinguish and identify chiral molecules or materials, specifically their enantiomers (mirror image isomers). Its principle and mechanism are mainly based on the asymmetry of chiral molecules in their spatial structure and their interaction with the chiral environment [150]. Although chiral molecules have the same chemical composition, they may exhibit significant differences in physical, chemical, and biological properties due to their different three-dimensional spatial arrangements (such as left-handedness and right-handedness). The core mechanism of chiral recognition depends on the diastereomeric interaction between chiral molecules and chiral selectors (such as chiral stationary phases, chiral reagents, or chiral catalysts) [151,152]. These interactions may include hydrogen bonding, π-π stacking, electrostatic interactions, hydrophobic interactions, and steric hindrance effects, resulting in different binding energies, reaction rates, or mobilities of the enantiomers in a chiral environment, to achieve separation and recognition.

Chiral materials play a key role in chiral recognition, analysis, and detection. For example, in chiral chromatographic analysis, chiral stationary phases achieve separation by selectively adsorbing chiral molecules; in chiral sensors, chiral materials (such as chiral metal–organic frameworks or chiral nanomaterials) generate signal responses (optical, electrochemical, or mass changes) through the specific recognition of chiral molecules [151,152]. In addition, chiral spectroscopy techniques (such as circular dichroism and vibrational circular dichroism) provide structural information and optical activity of chiral molecules by utilizing the different absorption properties of chiral molecules in response to left-handed and right-handed circularly polarized light [46]. Surface modification or functionalization of chiral materials can also enhance their selective recognition ability for specific chiral molecules. For example, chiral polymers prepared by molecular imprinting technology can simulate natural chiral recognition sites and efficiently detect target molecules [153,154].

#### 4.3.2. Research Status and Application

The development of chiral electrochemical sensors using chiral materials primarily relies on their ability to recognize chiral molecules and their specific electrochemical signal conversion function. First, materials with chiral structures or chiral recognition sites (such as chiral metal–organic frameworks, chiral polymers, chiral nanomaterials, or chiral carbon materials) are selected as the core recognition elements of the sensor. These materials enhance their selective binding ability to chiral molecules by surface modification or functionalization (such as introducing chiral ligands, chiral molecular imprinting, or chiral self-assembled monolayers) [150,151,152]. Then, the chiral material is fixed on the electrode surface (such as glassy carbon, gold, or printed electrode) to form a chiral-sensitive interface. When the target chiral molecule is in contact with the sensor, the chiral material selectively recognizes and binds the enantiomers through hydrogen bonds, π-π interactions, electrostatic interactions, or spatial matching mechanisms, resulting in changes in the electrochemical properties of the electrode surface (such as current, potential, or impedance) [152,153,154]. This change can be detected using cyclic voltammetry, differential pulse voltammetry, or electrochemical impedance spectroscopy to achieve high sensitivity and high selectivity in quantitative analysis of target chiral molecules. Chiral electrochemical sensors have broad application prospects in drug analysis, environmental monitoring, and biosensing [155,156].

Chiral electrochemical sensors have been widely used due to their advantages of simplicity, rapidity, low cost, and high sensitivity. Carbon nanotubes can be regarded as cylinders composed of graphite sheets, and their parameters determine their distinct structural morphologies. The single-walled carbon nanotubes can exhibit chirality when n = ∂ m = ∂ 0, and the short-diameter multi-walled carbon nanotubes prepared by chemical vapor deposition may also have chirality [157].

Niu et al. used the π-π interaction to encapsulate the guest C60 in the main lanthanide nanomaterial MOFs L/D-[La(BTB)] n. They successfully constructed a new chiral recognition composite material. Due to the host–guest interaction and the inhomogeneity of charge distribution, a significant electrostatic potential difference is generated in the chiral C60@[La(BTB)] n, resulting in a strong built-in electric field, thereby comprehensively improving the conductivity of the chiral material. This concept of enhanced charge separation through host–guest interaction creates an efficient chiral electrochemical sensor [27].

### 4.4. Chiral Materials for Electrocatalytic Reactions

Due to their unique structural symmetry-breaking characteristics, chiral materials show significant regulatory advantages in electrocatalytic reactions. The chiral center or helical structure can optimize the adsorption energy of the reaction intermediate on the catalyst surface by inducing asymmetry in the local electron distribution, thereby reducing the reaction energy barrier and enhancing catalytic activity [158]. For example, chiral metal–organic frameworks (MOFs) or chiral nanocarbon materials can selectively regulate reaction pathways by exposing high-density active sites and chiral microenvironments, such as promoting proton-coupled electron transfer in hydrogen evolution reaction (HER), or improving the selectivity of specific products (such as formic acid or ethylene) in carbon dioxide electroreduction [27]. In addition, the spin polarization effect of chiral materials may accelerate charge transport kinetics by affecting the electron spin state, and its stereo configuration can inhibit the poisoning or agglomeration of the catalyst surface, thereby enhancing stability. The design of these materials offers a novel approach to developing efficient and directional electrocatalytic systems, particularly in energy conversion and asymmetric synthesis [159,160,161,162].

Liang et al. [25] verified and evaluated the effect of chiral molecular functionalization on the OER activity of two-dimensional hybrid chiral/achiral molecules–transition metal oxide electrodes. This study demonstrates that helicene chirality significantly enhances the advanced OER catalyst activity via electron spin polarization at the surface. Chiral functionalization preserves the catalyst composition and enables co-optimization with methods like Fe doping, allowing electron spin catalysis to surpass the limitations of Sabatier scaling, as depicted in the volcano plot. Electrode structure comparisons guide rational optimization, paving the way for next-generation catalytic systems via molecular chiral engineering.

## 5. Application of Chiral Materials in the Field of Biology

### 5.1. Chiral Materials Are Used for Enantiomeric Separation

Chiral materials play a key role in the selective separation of enantiomers. The core principle is to use the diastereomeric interaction between chiral materials and enantiomers to achieve separation [163]. Chiral materials (such as chiral stationary phases, chiral membranes, or chiral adsorbents) have specific chiral recognition sites or chiral structures, which can produce different binding abilities to different enantiomers through hydrogen bonding, π-π interaction, electrostatic interaction, or steric hindrance [152,153,154]. For example, in chiral chromatography, chiral stationary phases can achieve separation by selectively adsorbing and desorbing chiral molecules, resulting in different distribution coefficients of enantiomers between the mobile phase and the stationary phase. In chiral membrane separation, the chiral membrane material selectively allows one pair of enantiomers to pass through the membrane pore. It blocks the other pair of enantiomers through the dual mechanism of size screening and chiral recognition of chiral molecules [164,165,166,167,168].

In addition, chiral metal–organic frameworks (MOFs) and chiral covalent organic frameworks (COFs) are also widely used in the adsorption and separation of enantiomers due to their highly ordered pore structure and adjustable chiral environment. The selective separation technology of chiral materials is significant in pharmaceuticals, chemical synthesis, and biotechnology, especially in the purification and production of single-enantiomer drugs [163,169,170,171].

For example, Narmadha et al. [163] successfully prepared homochiral COF nanochannel membranes for the selective separation of chiral amino acids by introducing a chiral center (L-phenylalanine methyl ester) into an organic ligand. The membrane displayed exceptional enantioselectivity toward racemic phenylalanine, achieving a maximum enantiomeric excess (ee) of 99.4%. Adsorption studies and molecular modeling revealed a preferential binding affinity for D-isomers over L-isomers, directly corroborating the experimental results [163,172,173]. The membrane demonstrated good stability and repeatability over three cycles, providing an essential reference for designing efficient chiral membranes.

### 5.2. Chiral Materials for Asymmetric Catalysis

Chiral materials play an essential role in asymmetric catalysis. The core induces enantioselectivity in chemical reactions through a chiral environment, enabling the efficient synthesis of a single chiral product [174,175]. Chiral materials (such as chiral metal–organic frameworks, chiral polymers, chiral nanomaterials, or chiral molecular sieves) can be used as catalysts or catalyst carriers to provide specific chiral active sites or chiral microenvironments. These materials interact with the reaction substrate through chiral ligands, chiral surface modification, or chiral pore structure, guiding the spatial orientation of the reaction transition state and preferentially generating a pair of enantiomers [176,177]. For example, chiral metal–organic frameworks (MOFs) can enhance the enantioselectivity of catalytic reactions by limiting the spatial arrangement of substrates through their chiral channels; chiral polymers or chiral nanoparticles achieve asymmetric induction through the specific binding of surface chiral sites to substrates. Applying chiral materials in asymmetric catalysis improves the efficiency and selectivity of chiral synthesis and reduces the formation of by-products. It has broad application prospects in pharmaceuticals, fine chemical synthesis, and materials science [169,170,171].

In 1893, Alfred Werner, the founder of modern coordination chemistry, first proposed that the six-coordinated transition metal complex has an octahedral configuration, and further confirmed that its central metal has chiral characteristics [178]. Since then, six-coordinated metal chiral complexes have been widely used in various fields, including medicinal chemistry, supramolecular chemistry, and catalytic science, for over a century. Despite its rich functions, the preparation methods for such chiral substances are still relatively limited, and research on synthesizing these substances by catalytic asymmetric methods is still in its infancy [179,180].

Since the beginning of the 20th century [181], synthetic chemists have been developing asymmetric catalytic strategies to efficiently and accurately synthesize chiral molecules. The early research focused on the construction of tetrahedral carbon-centered chiral molecules. In recent years, the research scope has gradually expanded to include the leading group element-centered chiral molecules, represented by boron, phosphorus, silicon, and sulfur [182,183,184]. It is worth noting that although transition metal elements occupy a broader chemical space in the periodic table of elements, the research progress of catalytic asymmetric synthesis of metal chiral compounds is significantly lagging, and this field needs to be further explored.

Based on the above background, Chu et al. [185] achieved a significant breakthrough in the asymmetric catalytic synthesis of metal–chiral molecules with high stereoselectivity (exceeding 90% ee) for the first time. Based on the kinetic resolution strategy, this study successfully achieved the efficient asymmetric synthesis of chiral iridium complexes by palladium-catalyzed asymmetric Suzuki–Miyaura cross-coupling reaction. This result offers a novel approach to the synthesis of chiral metal complexes.

### 5.3. Chiral Materials for Biomarkers

Chiral materials have critical applications in the detection and analysis of biomarkers. The core advantage of chiral materials is their high selective recognition ability for chiral molecules, which can effectively distinguish and detect biomarkers with chiral characteristics in vivo (such as chiral amino acids, chiral metabolites or chiral drug molecules) [186]. Chiral materials (such as chiral metal–organic frameworks, chiral nanomaterials, chiral polymers, or chiral molecularly imprinted materials) can form specific chiral recognition sites by surface modification or functionalization, which can specifically bind to the target chiral biomarkers through hydrogen bonding, π-π interaction, electrostatic interaction, or spatial matching [152,153,154]. For example, chiral fluorescent sensors can use the selective binding of chiral materials to chiral markers to cause changes in the fluorescence signal, thereby achieving high sensitivity detection; chiral electrochemical sensors convert chiral recognition events into electrochemical signals (such as current, potential, or impedance changes) by modifying the electrode surface with chiral materials for real-time monitoring of the concentration of biomarkers. In addition, chiral materials can also be combined with mass spectrometry, chromatography, or spectroscopy to enhance the efficiency of separating and identifying chiral biomarkers [187]. Chiral materials have great potential for application in disease diagnosis, drug metabolism research, and personalized medicine.

Metabolizing the chirality of small molecules is of great significance in regulating physiological processes and detecting human health [188]. Abnormal enantiomeric ratios of chiral molecules in biofluids and tissues are observed in many diseases, including cancer, kidney, and brain diseases. Therefore, chiral small molecules have broad application prospects in disease diagnosis, prognosis, adverse drug reaction monitoring, pharmacodynamics research, and personalized medicine. However, it remains challenging to achieve cost-effective and reliable chiral small molecule analysis in clinical operations, partly due to the wide variety and low concentrations of chiral small molecules.

Recently, Zheng et al. systematically addressed the identification and analysis of chiral biomarkers using diverse molecular measurement techniques. The study first outlined the correlation between chiral biomarkers and diseases, followed by the challenge of detecting low enantiomer concentrations in clinical settings. Working principles, merits, and limitations of different analytical methods were then analyzed. The discussion concluded with a discussion of challenges and prospects for achieving cost-effective, accurate enantiomer detection in biomedical research and clinical applications [28].

### 5.4. Chiral Materials Can Be Used for Disease Treatment

The application of chiral materials in disease treatment is primarily based on their efficient delivery, targeted recognition, controlled release of chiral drugs, and specific response to the biological environment [189,190]. Chiral materials (such as chiral nanoparticles, chiral metal–organic frameworks, chiral hydrogels, or chiral liposomes) can specifically interact with chiral molecules (such as proteins, cell membranes, or enzymes) in vivo through their chiral structure or chiral surface modification, thereby improving the targeting and bioavailability of drugs [191,192]. For example, chiral nanoparticles can selectively target diseased cells or tissues through a chiral recognition mechanism, thereby reducing side effects on normal cells. Chiral metal–organic frameworks (MOFs) can also be utilized as drug carriers, and their chiral pore structure can be leveraged to achieve controlled release and a long-term effect of the drugs. In addition, chiral materials can also achieve intelligent drug delivery by responding to external stimuli (such as pH, temperature, or enzyme activity), showing significant advantages in tumor treatment, inflammation regulation, and neurodegenerative disease treatment [193,194]. The application of chiral materials not only enhances the therapeutic effect of drugs but also provides new tools and strategies for precision medicine and personalized treatment.

The effect of chirality on immune response has attracted great interest in cancer vaccine research in recent years. However, studies on the effects of chiral synthetic peptide hydrogels as cancer vaccines and biomaterials in anti-tumor immunotherapy have rarely been reported [195]. Here, Ding et al. [196] demonstrated the key role of the chirality of peptide hydrogel residues in regulating anti-tumor immunity and local immune microenvironment. This study reveals that poly(γ-ethyl-D-glutamic acid)-based hydrogel (D-Gel) induces increased immune cell infiltration compared to its L-enantiomer counterpart (L-Gel). However, D-Gel promotes stronger inhibitory marker expression on antigen-presenting cells and exacerbates T cell exhaustion, establishing a local chronic inflammatory and immunosuppressive microenvironment that diminishes antitumor efficacy. In contrast, L-Gel elicits a host immune response leading to effective tumor suppression. The work highlights the role of residue chirality in modulating local immune microenvironments and shaping antitumor immune responses.

In 2024, Zhang et al. [197] engineered polypeptide TGGGPLGVARGK-GGC-induced chiral manganese dioxide superparticles (MnO_2_ SPs) for quantitative MMP-9 detection in vitro and in vivo. L-MnO_2_ SPs exhibited twice the binding affinity to CD47 receptors (cancer cell “don’t eat me” signaling protein) compared to D-MnO_2_ SPs. Surface-functionalized with an iRGD targeting ligand, these SPs accumulated in tumors, underwent MMP-9-triggered decomposition into ultrasmall NPs, and enabled efficient renal clearance. This chiral-engineered platform exhibits dual functionality, featuring CD47-targeted tumor accumulation and MMP-9-responsive degradation for theranostic applications.

## 6. Conclusions and Foresight

### 6.1. Research Progress and Main Achievements of Chiral Materials

Chiral materials have achieved remarkable research progress and essential results in many fields in recent years due to their unique structural asymmetry and chiral-dependent physical and chemical properties. In optics, chiral materials have achieved efficient circularly polarized light (CPL) emission and detection through mechanisms such as circular dichroism (CD), aggregation-induced emission chiral inversion (AIE-CI), and excited state chirality, which has promoted the development of circularly polarized organic light-emitting diodes (CP-OLEDs) and chiral perovskite light-emitting devices. In quantum science, the discovery of the chiral-induced spin selectivity (CISS) effect provides a new direction for research on spintronic devices, topological insulators, and quantum computing. The application of chiral materials in spin filtering, topological superconductivity, and high-temperature superconductivity has also made significant breakthroughs. In electronics, the ferroelectric effect, electromagnetic chiral anisotropy, and giant photovoltaic effect of chiral materials provide new design ideas for efficient energy conversion and memory devices. In biology, chiral materials have demonstrated broad application prospects in drug development, precision medicine, and biosensors through the selective separation of enantiomers, asymmetric catalysis, biomarker detection, and disease treatment.

### 6.2. Future Research Directions and Application Prospects of Chiral Materials

In the future, the research of chiral materials will develop in the following directions: Multifunctional integration and intelligence: By designing chiral materials with multiple functions (such as optical–electrical–magnetic multi-functional integration), intelligent responsive chiral devices are developed to realize real-time response and regulation to external stimuli (such as light, electricity, magnetism, temperature, pH, etc.). High-performance chiral optical devices: Further optimize the circularly polarized light emission and detection performance of chiral materials, develop efficient and stable CP-OLED, chiral perovskite light-emitting devices, and chiral plasma sensors, and promote the development of 3D displays, quantum communication, and biological imaging technology. Spintronics and quantum technology: In-depth study of the physical mechanism of the CISS effect, development of spintronic devices and topological quantum materials based on chiral materials, and promotion of the practical application of low-power spin memory and quantum computing technology. Chiral catalysis and green chemistry: Design efficient and highly selective chiral catalysts, promote the development of asymmetric synthesis and green chemistry processes, and reduce by-products and environmental pollution in chemical synthesis. Biomedical applications: Development of targeted drug delivery systems, biosensors, and disease diagnosis tools based on chiral materials to promote the development of personalized medicine and precision therapy. Exploration of new chiral materials: Through interdisciplinary cooperation, explore new chiral materials (such as chiral metal–organic frameworks, chiral covalent organic frameworks, chiral two-dimensional materials, etc.), and expand the application of chiral materials in energy, environment, and information science. In conclusion, research on chiral materials will continue to promote scientific and technological progress, providing new solutions to address significant challenges in energy, the environment, health, and information technology.

## Data Availability

No new data were created or analyzed in this study.

## Abbreviations

The following abbreviations are used in this manuscript:

CD	Circular dichroism
VCD	Vibrational circular dichroism
ROA	Raman optical activity
CPL	Circularly polarized light
LCPL	Left-handed circularly polarized light
RCPL	Right-handed circularly polarized light
CISS	Chiral-induced spin selectivity
SSE	The spin Seebeck effect
CPASS	Chiral-phonon-activated spin Seebeck effect
BPVE	Bulk photovoltaic effect
